# Coal Fires

**Published:** 1871-02

**Authors:** 


// HALL'S

### JOURNAL OF HEALTH.

Vol. XVIII.—FEBRUARY, 1871.—No. II.

### COAL FIRES.

Serious inconvenience to health is sometimes occasioned by tardiness in kindling a coal fire; passengers in railroad cars have often undergone incalculable sufferings from this cause.

Before coal kindles it must be heated through and through, made hot enough to blister the fingers in an instant, although still black. It is easy to see that a small bit of coal will get thus heated sooner than a larger one; hence the smaller the coal, the sooner will it ignite.

Coal must be kindled with wood. This wood will give out a certain amount of heat, and no more; and as a given amount of heat is necessary to kindle the coal, the more wood, and the less coal, and the smaller the pieces, the sooner and more certain will the fire be lighted.

In the face of these facts, persons are frequently seen in rail cars, when the fire in the stove is low, to put on a large amount of coal, the result being that the more coal put on, the more the fire will not burn, because the small amount of heat is distributed over a large amount of coal, all of which is heated some, but none of it heated enough for ignition.

The more a coal fire is stirred, if a little low, the more certain it is to go out.

The best way to replenish a coal fire is to put on a small amount of coal while it is burning well; and after this is thoroughly kindled, and has been red for a short time, add a little more coal. In this way a fire may be kept burning a whole day in a grate without using the poker once; and good housekeepers know that every time a poker is used, the ashes fly in every direction, and valuable time is expended in brushing them up. If a poker must be used, the time to do it is when fresh coal has been thoroughly kindled, for then there is no danger of its going out.

If a coal fire is burning too much, either cover it with some of the ashes which have fallen through the grate; this makes the mass more compact, and diminishes the draught, or if it is desirable to put the fire out altogether, as when going to bed, press the coal down from the top with a shovel or blunt-edged poker.

It has been the custom to use the largest-sized coal for the furnace; this requires a great waste of wood in kindling, besides much time is lost in firing up in the morning, the very time when most heat is wanted, and wanted quickly. It will take less coal, and give incomparably more comfort, to feed a furnace with coal, the largest piece of which is not larger than a hen's egg, only taking care to put on a little coal every hour. Observation and close calculating economy has shown this to all our river boats, tugs, and steamers.

As a well-warmed house and a brightly burning fire in the grate adds largely to the comfort, and life, and enjoyability of winter calls, these items about coal fires are seasonable. The most beautiful, the cheeriest, and the most healthful open fire known, is that which is furnished by the Low Down grate, made in Philadelphia by Dixon and Sons; it consumes no more coal than an ordinary grate, and gives out more heat by nearly two to one.

